# Operations on the Liver

**Published:** 1898-04-30

**Authors:** Leonard A. Bidwell

**Affiliations:** Senior Assistant-Surgeon to the Hospital


### Medical Progress and Hospital Clinics.

[*The Editor will be glad to receive offers of co-operation and contributions from members of the profession. All letters should be addressed to* The Editor, at the Office, 28 & 29, Southampton Street, Strand, London, W.C.

### OPERATIONS ON THE LIVER.

A Postgraduate Lecture, delivered at the West London Hospital by Leonard A. Bidwell, F.R.C.S., Senior Assistant-Surgeon to the Hospital.

(*Continued from page* 7.)

In the following four cases of cholecystotomy gall-stones were removed in two, and in the other two no gall-stones were found; but in one of these the trouble was due to adhesions which had formed in consequence of former gall-stones, while in the other case the cause of the dilatation of the gall-bladder is unknown.

Case X.: A man, aged 30, was admitted into the West London Hospital under Dr. Ball on account of attacks of severe abdominal pain, which were, however, unaccompanied by jaundice. The first attack had occurred two years previously, and had been so severe that the patient became unconscious. Two other attacks had occurred, but without jaundice. The pain lasted two days, and was acute for about twelve hours, and was relieved by morphia; it was accompanied by vomiting, shivering, and profuse sweating. No tumour could be felt as the patient was very muscular. The gall-bladder was exposed by an incision in the right linea semilunaris, and was found to be closely packed with stones. Some 212 stones were removed from the gall-bladder and cystic duct, and at the close of the operation bile flowed freely. The gall-bladder was stitched to the peritoneum and drained for ten days; bile continued to flow from the wound for two days longer, but the wound was quite healed in three weeks. The patient is still in good health.

Case XI.: Mrs. R., aged 35 years, was admitted into the West London Hospital under Dr. Seymour Taylor on account of several typical attacks of gall-stone colic, followed by jaundice. She had become emaciated. The gall-bladder was exposed by an incision in the right linea semilunaris, and was found to be dilated, and stones were felt in the cystic duct. After opening the gall-bladder about two ounces of mucus escaped, and 36 stones were extracted from the neck of the bladder and from the cystic-duct. One stone at the extremity of the cystic-duct could not be extracted, so was left. The gall-bladder was drained, but no bile escaped until the fourth day, after which it continued for five or six days, when the tube was removed. The patient made an uninterrupted recovery. The flow of bile showed that the stone had become dislodged, but had this not occurred it was intended to inject turpentine and olive oil to dissolve it.

In the two following cases no gall-stones were found:

Case XII.: F. I., aged 40, came to me from Valparaiso, suffering from what seemed to be typical gallstone colic. After consultation with Dr. Herbert Hawkins, cholecystotomy was performed, and the gall bladder found to be thickened and adherent to the omentum. The adhesions were divided, and the gall-bladder drained. The patient made a good recovery, and had no more attacks of pain.

Case XIII.: Mrs. E., aged 38, was sent to me at the West London Hospital on account of gall-stone colic, said to be followed by jaundice. Some swelling could be felt in the region of the gall-bladder, so cholecystotomy was performed, no stone was found, but the gall-bladder was found to be distended with mucus, and there were some adhesions about the cystic-duct. The gall-bladder was drained for a week. The patient made a complete recovery.

The other two operations on the gall-bladder need only be briefly referred to, as they are seldom performed. Cholecystectomy, or removal of the gall-bladder, is only needed in malignant disease, or when the cystic-duct is completely occluded; cholecyst-enterostomy, or the establishment of an artificial communication between the gall-bladder and the duodenum was formerly employed in cases of occlusion of the common duct by calculi, but now the calculi can be removed. The anastomosis has been successfully made several times by Murphy's button.

I now wish to draw your attention to the important and most modern class of operation on the biliary-ducts. In all operations for gall-stones when the gall-bladder and cystic-duct have been emptied of stones, the common bile-duct, as it runs between the layers of the lesser omentum, should be examined between the finger and thumb. By placing one finger in the foramen of Winslow, the bile-duct is easily explored as far as the duodenum. If a stone be impacted in the duct it may be pushed on into the intestines, or back into the gall-bladder, but if neither of these proceedings is possible, it may be crushed with a pair of dressing forceps, the blades of which have been guarded with drainage tubing (this operation is called choledocho-lithotrity), it may be needled and then crushed, or an incision may be made into the duct immediately over the stone, and this removed with forceps (this operation is called choledocho-lithotomy or, more simply, choledochotomy). After extraction of the stone, the incision in the duct should be closed with sutures if it is accessible; if not, the duct may be left open, and a rubber drainage tube or a gauze plug inserted down close to the wound in the duct. The gall-bladder must be stitched to the peritoneum and drained. A little bile usually escapes from the wound in the bile-duct for a few days, but quickly ceases if there be no stricture. Should it be impossible to get at the stone, or should the duct be completely obliterated by previous inflammation, the operation of choledocho-duodenostomy (*i.e.*, the formation of a new opening between the common bile-duct and the duodenum) may be performed. When a new growth affects the bile-duct, the whole duct may be removed (choledochectomy), the gall-bladder being afterwards united to the duodenum.

The last operation to which I will refer is one of the rarest of those performed on the bile-ducts. It is called hepaticostomy, and consists in the removal of gall-stones from one of the hepatic ducts in the portal fissure. The position of these ducts makes the operation one of great difficulty. A description of the following case will best explain the operation:—

Case XIV.: Mrs. G., aged 37, was brought to me by

Dr. Dickman, of Chiswick. One year previously she had her first attack of acute biliary colic, followed by jaundice; similar attacks recurred at short intervals, and four months before I saw her an exploratory laporotomy had been performed at another hospital, the gall-bladder being found to be empty, and no stone being felt in the cystic or common ducts. Two days after this operation she had another severe attack of biliary colic, and became jaundiced, and since that time she has not been free from pain for many days together. The patient was emaciated and jaundiced, her temperature was raised, and there was tenderness, but no tumour, in the region of the gall-bladder. The abdomen was opened in the old scar, and the omentum found to be adherent to the parietal peritoneum and to the gall-bladder. After dividing these adhesions, the gall-bladder, cystic and bile ducts were explored and found to be empty, but on passing the finger into the portal fissure the left hepatic duct was found to be dilated and filled with stones. As it was impossible to push these stones into the gall-bladder or into the common duct, an incision was made into the hepatic duct, and about twelve gall-stones extracted. Suture of this opening was, of course, impossible, so drainage tubes were placed down to the opening into the duct and into the gall-bladder; a gauze drain was also inserted. The patient, who was in a very feeble state before the operation, suffered from severe shock, accompanied by incessant vomiting, and never really rallied; she died forty-eight hours after the operation. The bile had escaped freely from the tube in the gall-bladder, and there were no symptoms of peritoneal irritation.

The only other condition which may demand operation is biliary fistula. This may be due either to gall-stones, malignant disease, or to actynomycosis. It may follow a cholecystotomy where the block of the common bile duct has not been relieved. The fistula is usually situated at the umbilicus, or in the right hyperchondrium. When the whole of the bile escapes by a biliary fistula, rather rapid emaciation ensues. If the condition is due to gall-stones, probably some of these will be found in the discharges; the treatment then consists in removing the cause by cholecystotomy, when the fistula will close. When the fistula is due to blockage of the common bile duct after cholecystotomy, either the operation of cholecystenterostomy may be performed, or the stones may be removed from the duct by choledectotomy. When due to malignant disease no operation is of any use.

### "ACCOUCHEMENT FORCÉ."

In this country "accouchement forcé" is a discredited operation. In his admirable little book on "Difficult Labour" Dr. Herman says: "The 'accouchement forcé,' which is sometimes recommended, means the rapid forcing open (that is, tearing open) of the os with the hand, separation of the placenta, turning, and rapid dragging away of the child, followed by the removal of the placenta. This is the most dangerous of all modes of treatment. Rigby cautioned against it. Müller rightly terms it a 'murderous practice.' Statistics show that about half the cases so treated die."

Other works either do not mention it or condemn it, and we may safely say it is a proceeding which at the present time hardly comes within the range of practical midwifery. It is then with no small interest that we see a paper in the New York *Medical Record*, by Dr. S. Marx, Assistant Physician to the New York Maternity Hospital, in which he tabulates fifty cases of accouchement forcé for various serious conditions, and strongly advocates its claims for recognition. The proceeding which he advocates is not a mere matter of digital or manual dilatation of a rigid os, but the induction of labour at a chosen time, often while the cervix is present; the manual dilatation of the os; the extraction of the child; and in many cases the manual removal of the placenta.

Dr. Marx says: "There seems to be, even to this day, an inherent fear in the minds of some, who are otherwise clear and shrewd observers, that the elective manual dilatation of the pregnant uterus is fraught with such danger that the woman, not to mention the unborn fœtus, is destined to almost certain death," and there can be no doubt that this pretty fairly expresses the opinions of many practitioners.

Let us, then, see what Dr. Marx is actually doing. "While I have in no single case of the series of 50 here reported attempted to operate against time, yet occasionally a case would arise which would necessitate rapid work. . . . For example . . . a grave case of eclampsia in a primapara at the end of the eighth month. No labour pains; a cervix present nearly an inch long. Yet within 17 minutes I had fully dilated the cervix, perforated the first baby, which was dead, turned the second living baby, manually removed the placenta, and tamponed the utero-vaginal tract. She made a beautiful recovery." Another case was one of "complete placenta prævia in a V para, with an extremely succulent lower uterine zone and a long cervix present. The uterus was dilated, twins were delivered by version, the placenta was removed manually, and the uterus tamponed in a trifle less than seven minutes. Aside from a temporary pyrexia everything went along smoothly. Both these cases were timed by the physicians present." We have quoted the above so as to show that what is done by Dr. Marx is really what has been so strongly condemned under the name "accouchement forcé." Now for the results. In the table given there are fifty cases, many of them cases of great severity, and three deaths. Really, however, there were four deaths among the fifty cases, for in one on whom the operation was done for ante-partum sepsis laparotomy was afterwards performed, and the patient died.

Of the three cases returned as having died, in one it was found impossible to dilate with the fingers, and Dührssen's incisions were made; in another the patient was suffering from grave scarlet fever, and "was practically moribund" when labour pains started. This case, as the author says, ought never to have been subjected to an operation. In the third case the patient was suffering from uræmia, and died eighteen hours afterwards. Among the cases operated on were eight of placenta prævia, four of which were completely centrally implanted. "Result: Eight mothers—eight alive; nine children—all born alive, and when viable all lived (one mother had twins)." We should add to this number one case more, viz., the one which died, in which dilatation failed and Dührssen's incisions were employed.

Of uræmia or the eclamptic state there were 13 cases. "Result: Thirteen mothers—12 alive, one dead; 15 children—14 born alive, and when viable all lived (two mothers had twins, one of them dead ante partum, as certified to by the hand in the uterus, and accordingly perforated)."

Of course, accidents were met with. One case of deep laceration of the cervix, and one of a large vesico-vaginal fistula, are mentioned These, however, do not seem to have greatly troubled Dr. Marx. In the first, "the woman was placed on her back; her uterus was artificially prolapsed by bullet forceps and pressure from above; the cervix was delivered through the vulva, and sewed up as readily as though the condition was a lacerated perineum." In the other, "permanent catheter in the bladder and constant irrigation cured this nasty complication." We do not for a moment think that the experience of Dr. Marx will rehabilitate accouchement forcé in the eyes of the profession, except in quite exceptional cases, for, if a rapid emptying of the uterus is necessary, other means are available. But country practice is full of strange emergencies. A man may be caught in the midst of his rounds by a case of placenta prævia or eclampsia when he is far from help and from his instruments, and has nothing but his hands to work with, and it is well to be reminded how much can be done with them alone, and with what comparative safety so long as care is taken with antiseptics. It should be noted, however, that when there was no special urgency, and where the cervix was present, Dr. Marx made use of a preliminary tamponing of the cervix with strips of gauze, by means of which it was sufficiently dilated to admit "one or two fingers."

